# Applying Aluminum–Vertically-Aligned Carbon Nanotube Forests Composites for Heat Dissipation

**DOI:** 10.3390/nano9050758

**Published:** 2019-05-17

**Authors:** Yan-Rui Li, Chih-Chung Su, Shuo-Hung Chang

**Affiliations:** Department of Mechanical Engineering, National Taiwan University, Taipei 10617, Taiwan; d00522001@ntu.edu.tw (Y.-R.L.); r92522629@ntu.edu.tw (C.-C.S.)

**Keywords:** vertically-aligned carbon nanotube, thermal sheets, heat dissipation efficiency, natural cooling, chemical vapor deposition

## Abstract

Vertically-aligned carbon nanotube forests (VACNTs) with excellent axial heat dissipation properties were formed on aluminum foil to dissipate heat. In addition, the heat dissipation efficiency of aluminum–VACNTs composites in this work was compared with that of commercially available mainstream thermal sheets under the same natural cooling conditions. Chemical vapor deposition (CVD) was employed as a synthesis method using a three-segment high-temperature furnace. Subsequently, the temperature changes in a heating body with the aluminum–VACNTs composites was measured over time subject to natural cooling. In addition, the performance was compared with copper and pyrolytic graphite sheets. The experimental results revealed that the heat dissipation efficiency of the flexible aluminum–VACNTs composites was higher than that of clean aluminum foil, a copper sheet, and a pyrolytic graphite sheet by up to 56%, 40%, and 20%, respectively. Moreover, this work also verified the height of the carbon nanotube (CNT) did not influence the heat dissipation efficiency, indicating that the time cost of synthesis could be reduced.

## 1. Introduction

Because of the increasing performance and miniaturization of electronic components, an electronic component can emit a high amount of heat per unit area. Thus, heat dissipation is vital to maintaining the stable working condition of many electronic components [1,2,3,4]. Studies have indicated that heat dissipation solutions account for more than 50% of the total cost of high-power devices. Unless heat dissipation materials are found that are markedly more effective than the current standards, costs for solving heat dissipation problems will continue to increase [5,6]. Ever since the discovery of carbon nanotube (CNT), they have been known for their high thermal conductivity [7,8]. To date, numerous studies have explored the excellent thermal conductivity of isolated CNT and applied their findings to thermal interface materials (TIMs) [9,10,11,12,13,14,15,16]. However, the thermal conductivity depends on CNT directionality. Axial and horizontal thermal conductivity values can differ greatly [17]. When a synthesized CNT is transferred to a target to facilitate heat dissipation, the surface irregularity of the CNT composites and its arrangement can excessively reduce the contact efficiency, thereby reducing the overall heat dissipation capacity. Accordingly, a CNT must receive post-treatment before use. However, such a procedure is cumbersome and thus unsuitable for commercial applications. In some studies, CNT composites have been fabricated into thermally-conductive polymer composites as an alternative to TIMs. The composite materials are selected because of their light weight, thin volume, excellent corrosion resistance, and ease of processing, making them suitable for replacing currently used metal-finned tubes. However, numerous reports have discovered that the heat dissipation capability of polymer/CNT highly deviates from the theoretical values [18,19,20,21,22,23]; for the same reason such values deviate for TIMs. In other words, both types of materials suffer from problems related to CNT alignment and contact thermal resistance. In CNT alignment, Burg et al. [24,25] made important progress in relation to type-selective device integration of individual single-wall CNT.

In the present work, vertically-aligned carbon nanotube forests (VACNTs) were directly synthesized with aluminum foil. By adjusting the manufacturing parameters, the VACNTs exhibited excellent alignment properties with high densities. Subsequently, the aluminum–VACNTs composites were directly applied to a hot-working element to skip the steps required for straightening the arrangement of the CNT during the second round of assembly, and to avoid problems related to contact thermal resistance between different interfaces. Lastly, we compared our results for heat dissipation with those of pyrolytic graphite sheets commonly adopted in current high-end products and with those of copper sheets used in mid-end products. The results confirmed the strength of the method used in this work.

## 2. Materials and Methods

### 2.1. Aluminum–VACNTs Composites Synthesis

**Figure 1:**
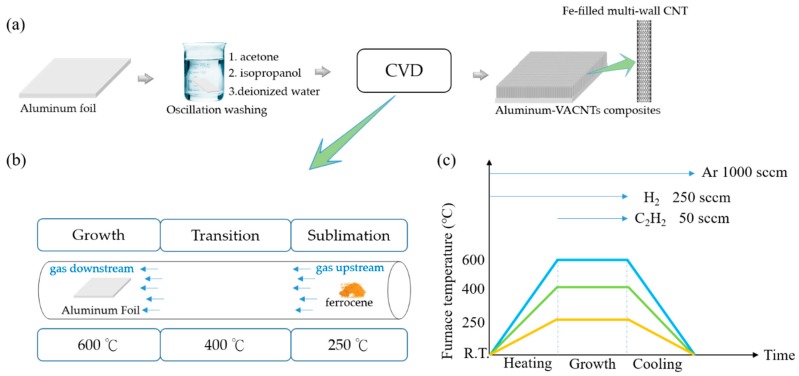
(**a**) Fabrication procedure of aluminum vertically-aligned carbon nanotube forests (VACNTs) composites; (**b**) synthesis setup in this experiment employing three-segment chemical vapor deposition (CVD); (**c**) recipes for the furnace and gas flow.

**Figure 2:**
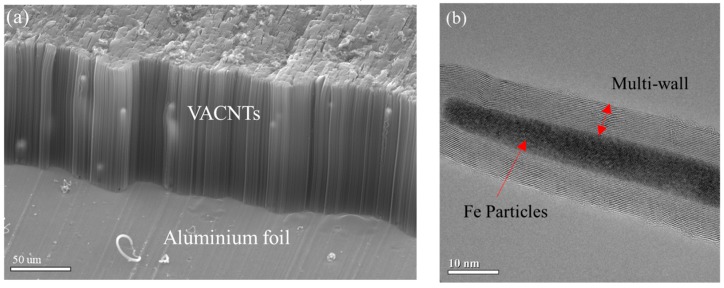
(**a**) Scanning electron microscopy (SEM) image of the aluminum–VACNTs composites from a 45° angle; (**b**) transmission electron microscopy (TEM) image shows Fe particles along the length of the carbon nanotube (CNT).

The floating catalyst method was employed to synthesize aluminum–VACNTs composites. This method is simple, does not require high-cost coating equipment, and can substantially reduce production costs compared with other methods that require the evaporation or sputtering deposition of a catalyst on the substrate. In the experiment, a three-segment high-temperature furnace was used for CVD to synthesize VACNTs in a 3-inch quartz tube under 1 atm. The furnace was divided into three compartments whose temperatures could be controlled independently, namely sublimation, transition, and growth compartments. Each compartment had its own independent heater and thermocouple. The temperatures for the three compartments were set at 250, 400, and 600 °C, respectively. Glass fiber blankets were used to separate the compartments to maintain each compartment’s own temperature. The substrate used was commercially-available household aluminum foil, which was washed to remove surface impurities by using acetone, isopropanol, and deionized water in that order. The cleaned aluminum foil was placed on a quartz plate and then in the growth compartment. A quartz boat with ferrocene (Merck, Darmstadt, Germany) was first placed outside the sublimation compartment; once the reaction began, the quartz boat was pushed into the sublimation compartment to commence the CNT synthetization reaction. When the ferrocene was heated past 400 °C, iron and carbon atoms were produced through pyrolysis. During the VACNTs manufacturing process, the iron atoms served as catalysts and the carbon atoms became a part of the carbon sources for forming the VACNTs. When the temperature began to increase, Ar at a flow rate of 1000 sccm and H_2_ at a flow rate of 250 sccm were sent through the quartz tube. The temperature increase duration was set to 20 min to ensure all three compartments reached the designated temperatures. Next, 50 sccm of C_2_H_2_ was sent through and ferrocene was pushed into the quartz tube to sublimate and follow the gas flow to the growth compartment for the synthetization reaction, as shown in Figure 1. The reaction time was adjusted according to the required height of the VACNTs. Figure 2a displays aluminum–VACNTs composites that had synthesized for 15 min, which were photographed by using scanning electron microscopy (SEM; JEOL JSM-6390, Tokyo, Japan at 15 kV). As shown in the figure, the VACNTs on the aluminum foil exhibited excellent growth density and alignment. Figure 2b presents a photograph obtained through transmission electron microscopy (TEM; JEOL JEM-2100F, Tokyo, Japan at 200 kV), revealing that the as-grown VACNTs were a type of Fe-filled multi-walled CNT. The CNT were filled with iron atoms.

### 2.2. Heat Dissipation Setup

The lumped-heat-capacity theory was adopted to calculate the heat change rate inside of a copper block [26,27]. The heat change inside the copper block could be calculated based on the measured temperature of the sample in the experiment. In the lumped-heat-capacity system, the heat was transmitted outwards from the inside of the copper block. The only difference was the different samples, with all other conditions remaining identical. The heat change rate (q) inside the copper block can be calculated as in Equation (1):

(1) $$q=-C\rho V\frac{dT}{dt}$$

where *C* denotes the specific heat (Jkg^−1^K^−1^) of the copper block, ρ denotes the density of the copper block, and *V* denotes the volume of the copper block.

**Figure 3:**
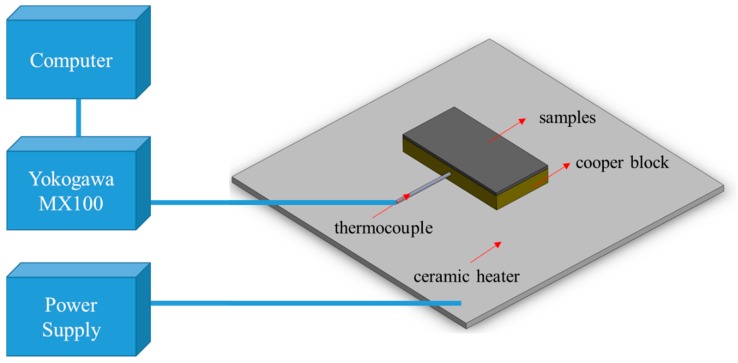
Experimental setup of heat dissipation measurement for different samples.

To measure the heat dissipation effects of the various samples on a high-temperature component, each sample with an area equal to the top surface was adhered to the copper block, which was heated to a high temperature and left to cool naturally. A ceramic heating plate was selected as the heating source alongside a power supply to provide energy. After the temperature of the copper block was higher than room temperature by 150 °C, the copper block was left to cool naturally. We considered this temperature sufficient for covering the working temperature range of most electronic components. Before we commenced cooling and recorded the temperature, the ceramic heater was replaced with a well-insulated fiberglass blanket to prevent the system from being influenced by the temperature of the ceramic heater. The temperature-cooling curve was also recorded at the mean time. Regarding temperature measurement, this work used a thermocouple temperature measurement system (Yokogawa MX-100, Tokyo, Japan), which employed a Type T thermocouple to measure temperature at a precision of 0.1 °C per second and was connected to a computer in order to record temperature change over time. Because the data points were highly dense, a mark was shown every 100 s to facilitate observation. When conducting the experiment, the whole system was placed in a closed environment without ventilation to ensure that the only independent variable was the different samples. This process is illustrated in Figure 3.

## 3. Results and Discussions

**Figure 4:**
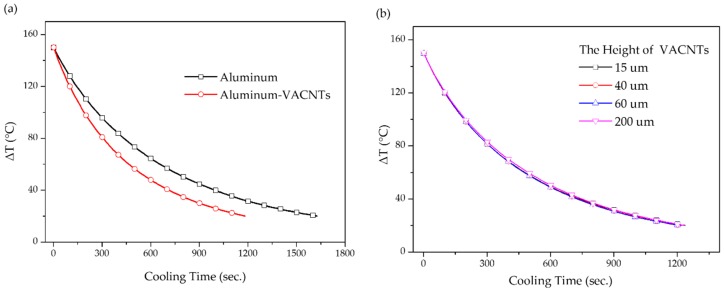
(**a**) The temperature–time curve for aluminum foil with and without VACNTs subject to natural cooling; (**b**) the temperature–time curve when employing various heights of VACNTs subject to natural cooling.

First, we determined whether VACNTs contributed to heat dissipation. Because this work employed aluminum–VACNTs composites materials, and aluminum foil has a horizontal heat-spreading effect, we first compared samples with and without VACNTs on aluminum foil. As shown in Figure 4a, the aluminum–VACNTs composites exhibited a shorter cooling time than did aluminum foil on its own. The amount of time required by the two types of materials to reach a certain temperature differed by approximately 450 s. A preliminary judgment, the VACNTs had an obvious positive-effect on heat dissipation. Notably, the final data point and the environmental temperature differed by 20 °C. From the perspective of thermal equilibrium, the larger the difference between the final data point and the environmental temperature is, the faster the cooling speed becomes; by contrast, a smaller difference indicates a slower heat dissipation speed. Therefore, this work considered that measuring the room temperature from 150–20 °C was sufficient for observation.

After confirming that employing VACNTs improved heat dissipation efficiency, this work examined whether the height also affected efficiency. Four heights of VACNTs were prepared for testing. As the results in Figure 4b show, the height of the CNT did not influence heat dissipation efficiency. This result indirectly confirmed the anisotropic thermal property [17]. Increasing the height of the VACNTs means that the contact area between the lateral area and the air increases. However, the lateral thermal conductivity of the CNT was not superior to the regular conductivity. Therefore, increasing the lateral area does not substantially improve their overall heat dissipation efficiency. In addition, previous studies have indicated that when the length of a CNT is within approximately 2 µm (i.e., shorter than the phonon mean free path), the thermal conductivity coefficient reduces multifold as the length decreases. As the length increases close to the phonon mean free path, the influence of its height on the thermal conductivity coefficient becomes minimal [28,29,30]. In the present work, the height of VACNTs far exceeded 2 µm, confirming that the increased height had no influence on thermal conductivity. In terms of commercial consideration, the time costs required for synthesizing CNT for use in thermal sheets can be substantially reduced. In addition, for electronic components with spatial limitations that require heat dissipation, reduced-height aluminum–VACNTs composites can render a thermal sheet thinner and more applicable while maintaining the same heat dissipation efficiency.

**Figure 5:**
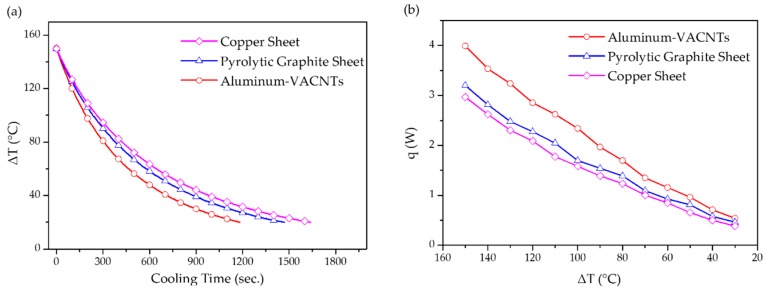
(**a**) The temperature–time curve for the aluminum–VACNTs composites, pyrolytic graphite, and copper sheets subject to natural cooling; (**b**) relation of temperature change versus heat change for the aluminum–VACNTs composites, pyrolytic graphite, and copper sheets subject to natural cooling.

This work subsequently compared the produced aluminum–VACNTs composites with commercially available mainstream thermal sheets (i.e., a graphite sheet and copper sheet). As shown in Figure 5a, the results revealed that the aluminum–VACNTs composites was superior in terms of heat dissipation. Notably, rather than a natural graphite sheet with inferior heat dissipation properties, the graphite sheet used in the present work was a pyrolytic graphite sheet with a high thermal conductivity coefficient, which is currently used in numerous commercial products. Equation (1) was adopted to calculate the rate of heat change inside the copper block (Figure 5b). By calculating the mean value of q for each sample, we discovered that the heat dissipation efficiency of the aluminum–VACNTs composites was higher than that of the copper sheet and graphite sheet by 40% and 20%, respectively. This is attributable to how the aluminum–VACNTs composites transmitted heat in three directions: The aluminum foil, itself, provided satisfactory horizontal heat transmission; subsequently, the VACNTs exhibited excellent heat transmission along the z-axis direction, thereby leading to rapid convection between the heat and surrounding air and causing heat dissipation. By contrast, the copper sheet and graphite sheet could only transmit heat in the plane direction. When the hot area was equal to the thermal sheet, the copper and pyrolytic graphite sheet had difficulty dissipating heat because the temperature was the same in each point, which meant that the heat could not be transmitted to other areas. Thus, they could only passively exchange heat with the surrounding air. In addition, the VACNTs not only exhibited superior axial heat dissipation but also demonstrated a heat radiation rate reaching 0.98–0.99 [31,32]. Accordingly, a portion of thermal energy can be converted to infrared radiation to facilitate heat dissipation. This indicates that the proposed composites are capable of thermal conduction as well as heat dissipation through thermal radiation.

## 4. Conclusions

In this study, a three-direction thermal sheet fabricated with aluminum and VACNTs was compared with pyrolytic graphite and copper sheets. The results confirmed that the aluminum–VACNTs composites exhibited more favorable heat dissipation, and the height of VACNTs did not affect the heat dissipation efficiency because of the anisotropy thermal property. The proposed method provides a facile and flexible system that enables effective thermal dissipation. As ever-smaller electronic devices are being produced, spatial restrictions of related applications will become more and more demanding. Accordingly, aluminum–VACNTs composites will be in greater development than other types of thermal sheets.
